# Asthma Length of Stay in Hospitals in London 2001–2006: Demographic, Diagnostic and Temporal Factors

**DOI:** 10.1371/journal.pone.0027184

**Published:** 2011-11-02

**Authors:** Ireneous N. Soyiri, Daniel D. Reidpath, Christophe Sarran

**Affiliations:** 1 School of Public Health, University of Ghana, Accra, Ghana; 2 Global Public Health, School of Medicine & Health Sciences, Monash University, Sunway Campus, Malaysia; 3 Met Office, Exeter, United Kingdom; University Hospital Freiburg, Germany

## Abstract

Asthma is a condition of significant public health concern associated with morbidity, mortality and healthcare utilisation. This study identifies key determinants of length of stay (LOS) associated with asthma-related hospital admissions in London, and further explores their effects on individuals. Subjects were primarily diagnosed and admitted for asthma in London between 1^st^ January 2001 and 31^st^ December 2006. All repeated admissions were treated uniquely as independent cases. Negative binomial regression was used to model the effect(s) of demographic, temporal and diagnostic factors on the LOS, taking into account the cluster effect of each patient's hospital attendance in London. The median and mean asthma LOS over the period of study were 2 and 3 days respectively. Admissions increased over the years from 8,308 (2001) to 10,554 (2006), but LOS consistently declined within the same period. Younger individuals were more likely to be admitted than the elderly, but the latter significantly had higher LOS (p<0.001). Respiratory related secondary diagnoses, age, and gender of the patient as well as day of the week and year of admission were important predictors of LOS. Asthma LOS can be predicted by socio-demographic factors, temporal and clinical factors using count models on hospital admission data. The procedure can be a useful tool for planning and resource allocation in health service provision.

## Introduction

Globally, the morbidity and mortality associated with asthma places a high burden on health care infrastructure and services [1], [2], [3], [4], [5]. Asthma is a chronic condition which in the United Kingdom alone affects over 5.2 million people including 1.1 million children [6]. The UK together with the Republic of Ireland have the highest prevalence of asthma in the world [3]: it is the leading cause of hospital admissions particularly among children [7], and disproportionately affects certain ethnic groups and demographics [2]. The total cost of asthma is estimated to be £2.5 billion in the UK and Ireland and results in millions of lost working days [3]. Notwithstanding a significant literature on asthma and its impacts, most of the researches are focused on the clinical presentation of the disease.

Length of stay (LOS) refers to the duration of a hospital admission (i.e. the difference in days between the date of admission and the date of discharge). It reflects several aspects of hospital care including the complexity of the case, the efficiency of hospital care, and the nature of hospital policies on admission and discharge [8], [9], [10], [11], [12], [13], [14]. LOS can be used as an indirect estimator of resource consumption and efficiency within the settings of a hospital, and has direct implications for overall healthcare planning and policy [5], [9], [12].

Analyses of length of stay associated with asthma, and its correlates with demographic, hospital, and temporal factors are relatively unusual [7], [15], [16]. The standard procedures for decision making in the case of asthma hospitalizations vary widely during its diagnosis; but this ultimately affects the variability associated with its management in relation to the LOS [17], [18], [19].

The study proposed by Arnold and colleagues, which sought to identify the key clinical predictors for acute asthma exacerbations in paediatric patients [18], may also identify the clinical determinants of LOS. This study however has a sharp clinical focus. Meanwhile, investigations on the combined demographic, diagnostic and temporal determinants of asthma LOS in large populations are not common.

Some other earlier studies on LOS for asthma hospitalisation in the UK (Scotland) focussed on the trends in asthma admissions and how changes in hospital bed occupancy could measure resource use [5]. In that study, and others similar to it [20], [21], [22], they described the trends in the percentage change in asthma admission rates with respect to temporal and demographic explanatory variables (e.g., year of admission, aggregate ages and gender). Such studies have, however, not been sufficient in predicting or explaining the LOS for asthma sufferers, whilst accounting for variations in demographic and temporal factors.

The aim of this study was to extend earlier work by examining the independent effect of demographic, hospital, and temporal factors associated with asthma LOS, using hospital admission records from London (2001–2006).

## Methods

### Data

This study involved a secondary analysis of hospital administrative data from London, England. The data covered 56,832 emergency asthma hospital admissions from January 1^st^, 2001 to December 31^st^, 2006. After removing data with missing patient sex, 56,768 admissions were available for analysis. The data relate to 40,359 unique individuals.

Data were sourced from the nationally recorded Hospital Episode Statistics (HES) maintained by the National Health Service, England [23]. HES data do not include all fields for which individual hospitals may collect data, such as which attending staff were involved in patient management; and not all data were readily available for secondary analysis, such as any variation post-admission in the diagnosis.

Asthma admissions were defined as any hospital admission with a primary diagnosis of asthma; i.e., an International Classification of Diseases (ICD-)10 code of J45. The mis-coding rate of J45 is not known, although it is known that J45 and ICD-10 coded J46 (*status asthmaticus)* admissions do have a diagnostic overlap [24]. Unfortunately, the J46 data were not available. Previous research has indicated that the J45 coded admissions in the UK cover around 82% of all asthma admissions. Furthermore, in absolute numbers, there are more, severe cases among the J45 coded admissions than among the J46 admissions [24]. The implications of this are discussed further, towards the end of the article.

The length of stay was estimated as the difference in days between the date of admission and the date of discharge. All stays of less than 24 hours (i.e., <1 day) were recorded as zero days admission. This has implications for the underestimation of LOS, and is discussed later in the article. LOS is the total length of stay without regard to whether a patient may remain in hospital for reasons unrelated to asthma. It is, thus, a measure of the length of stay, for all causes, given admission for asthma.

Following other research,[2] solely for the presentation of descriptive data, LOS was categorised into short (less than 24 hours), medium (1–3 days), long (4–7 days) and very long (more than a week) stays.

The explanatory variables from the HES dataset that were included in the analyses could be broadly described as demographic (age, sex, ethnicity), hospital (primary diagnosis, secondary diagnosis, method of admission/discharge) and temporal (day of week, season and year of admission) variables. Data on sex (male and female) were used as recorded in the dataset. Age was categorised as: 0–4, 5–14, 15–44, 45–59, 60–74 and more than 75 years. The approach to age categorization in asthma research is not consistent; however, the categories used here account for differences between younger and older children as well as young adults and older adults. Ethnicity was represented by five categories of (i) White (-Irish, -British, -Any other white), (ii) Black (all black i.e. -African, -Caribbean, -American), (iii) Asian (Indian, Pakistani, Bangladeshi, Chinese), (iv) Mixed and (v) Unknown/not indicated. This grouping conforms broadly to the categories used by the Office of National Statistics, but collapses some of the categories in which there were small numbers [25].

The ICD-10, J45 diagnosis of asthma is sub-coded into four categories; however, 95% of all admissions fell within a single category (J45.9 “Asthma, unspecified” on their hospital records). Notwithstanding the uncertainty about the accuracy of the sub-coded diagnoses, primary diagnosis was retained as a separate explanatory variable in the model [16]. All the secondary diagnoses were categorised according to their respective ICD-10 codes and were included in the analysis. These included: (i) Acute upper respiratory infections; (ii) Other diseases of upper respiratory tract; (iii) Influenza and pneumonia; (iv) Other acute lower respiratory infections; (v) Suppurative and necrotic conditions of lower respiratory tract; (vi) Chronic lower respiratory diseases; (vii) Lung diseases due to external agents; (viii) Other respiratory diseases principally affecting the interstitium; (ix) Other diseases of pleura; (x) Other diseases of the respiratory system; (xi) Other non-respiratory system diseases; and (xii) Missing values. The categories viii – x of the secondary diagnosis, which had very few counts were grouped and reclassified as one category “Other diseases of the respiratory system”.

Some of the key derived variables created for the analysis included “day of the week”, “season”, and “year of admission”. The meteorological seasons were Spring (1^st^ March–31^st^ May), Summer (1^st^ June–31^st^ August), Autumn (1^st^ September–30^th^ November) and Winter (1^st^ December–February end). The year of admission was extracted from the date of admission.

### Data Analysis

Length of stay was modelled using negative binomial regression, with a random effect to take account of the lack of independence between admissions associated with repeat admissions of the same patient. Poisson regression is generally well suited for modelling count data. A negative binomial model, however, is preferred when there is over dispersion; that is, when the mean and the variance are not equal. In the context of LOS, this can occur if there are more 0 days of admission than anticipated under a Poisson model [26], [27], [28], [29]. This was formally tested using the likelihood approach suggested by Long and Freese [30].

For the expected LOS, the negative binomial regression can be presented in the form:
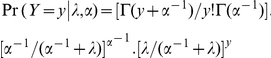
Where:

λ is the mean of the distribution;

α is the over dispersion parameter;

y is the LOS component represented by 0, 1, 2, …;

Γ is the gamma function.

A positive coefficient in the regression output indicates that a factor will increase the LOS relative to its reference category and conversely a negative coefficient will decrease the LOS relative to its reference category. The exponent of the coefficient can be interpreted, all other things being equal, as the proportionate increase (for values greater than 1) or decrease (for values between 0 and 1) of LOS associated with a one unit increase in the explanatory variable [29].

A positive coefficient in the regression output indicates that a factor will increase the LOS relative to its reference category and conversely a negative coefficient will decrease the LOS relative to its reference category. The exponent of the coefficient is a ratio and it can be interpreted, all other things being equal, as the ratio of length stay at one level of the covariate to length of stay at one level less than this on the covariate [29].

In the first instance, a full model with all explanatory variables was developed. This then led to a reduced model that excluded month of birth. The improved fit of the reduced model was established by the reduction in the Akaike Information Criterion (AIC) [31], [32], [33].

All analyses were conducted using Stata SE version 10.1 statistical packages (Stata Corporation, Texas, USA). Exemption from ethical review for the secondary analysis of hospital administrative data was obtained from the Monash University Human Research Ethics Committee (Number: 2011001092).

## Results

The median recorded length of stay (LOS) following admission was 2 days with a mean LOS of 3 days (sd = 6 days). The distribution had a heavy right tail with a range of 0–367 days. The majority (56.7%) of all admissions related to a person being admitted only once during the period 2001–2006; 17.4% of admissions were associated with a person being admitted twice, and 8.2% were associated with a person being admitted three times. This percentage declined rapidly, although one person was admitted 77 times during the period.

Table S1 shows the distribution of admissions across the key explanatory variables. Males and females each had close to 50% of all asthma admissions. Nearly half of all admissions were whites; with the other ethnic groups each contributing about a tenth. Meanwhile a fifth (20%) had no record of their ethnicity in the dataset. Most admissions were made directly through the hospitals' Accident and Emergency (A&E) departments (92%), and most discharges from hospitals were based on clinical advice or clinical consent (97%). The great majority of admissions were diagnosed as “Asthma, unspecified” (94%). The autumn months recorded the most admissions (30%) and the least were recorded during the summer months (22%). From 2001 to 2006 the number of admissions steadily increased – 27% over the 6 year period. This increase was well in excess of the 5.9% growth in the population that occurred in London between 2001 and 2009 [34].

In the multivariable analysis of the explanatory variables, a full model was developed that included all explanatory variables (Table S2). Month of birth showed weak effects, with only one month significantly different from the base month. A reduced model, removing month of birth as an explanatory variable was developed. The reduced model was retained on the basis of an improved AIC (239405.5 in the full model compared with 239394.8 in the reduced model), and little variation in the parameter estimates of the remaining explanatory variables.

All other things being equal, Females had a slightly longer LOS than males (1.11 times longer; 95%CI: 1.09–1.13). The association with age and LOS increased monotonically, with each age category over 0–4 years of age having a significantly longer LOS, from 1.07 (95%CI: 1.04–1.11) times longer for 5–14 year olds up to 3.43 times longer (95%CI: 3.31–3.55) for those over 75. The confidence intervals also suggest a fairly clear separation across the age groups, although this was not formally tested. All other things being equal, there was a small, but significantly longer LOS associated with being Black (1.05; 95%CI: 1.02–1.08) compared with White, and a slightly shorter LOS associated with having no recorded ethnicity (0.93; 95%CI:.91–.95). A primary diagnosis of “Predominantly Allergic Asthma” was associated with a significantly shorter LOS than the most common diagnosis of “Asthma, unspecified” (0.83, 95%CI:.79–.87). A number of the secondary diagnoses were associated with a longer LOS than those with an acute upper respiratory tract infection. Influenza, pneumonia, or other respiratory diseases, were associated with an increased LOS between 1.3 and 1.7 times longer.

Method of admission was significantly associated with LOS. Compared with admission through attendance at Accident and Emergency, referral by a General Practitioner was associated with a shorter LOS (0.90; 95%CI: 0.86–0.93), while referral by a Consultant Physician was associated with a significantly longer LOS (1.20; 95%CI: 1.12–1.28). All other things being equal, days of the week except Saturday were associated with a significantly longer LOS than Sunday admissions. Monday to Friday admissions were associated with an LOS about 1.31 times longer; Saturdays were associated with an LOS 1.09(95%CI: 1.05–1.13) times longer. Autumn, Winter, and Spring were all significantly associated with slightly longer (1.04 to 1.07 times longer) LOS than Summer.

Though the number of yearly admissions increased over the time period, the length of stay consistently reduced over the same time, with every year after 2002 being associated with a significantly shorter LOS. All other things being equal, patients admitted in 2006 had an LOS 0.71 times as long as someone admitted in 2001 (95% C.I. 0.68–0.73).

## Discussion

Asthma hospital admissions and their associated lengths of stay place a substantial burden on the health services, carers, and individual asthma sufferers [1], [3], [5]. Between 2001 and 2006, there were 56,832 asthma admissions in London associated with 40,359 individuals, accounting for around 170,500 days of hospitalised care; a finding consistent with earlier research [20], [35], [36]. The number of hospital admissions increased from 8,308 to 10,554 between 2001 and 2006, while the actual length of stay associated with each admission reduced significantly.

All the demographic, hospital, and temporal factors investigated were found to have statistically significant associations with the length of stay. The statistical association, however, does not necessarily translate into what might be regarded as variations in the length of stay with significant clinical impact. All other things being equal patient sex, ethnicity, primary diagnosis, and season of admission resulted in no more than a 10% variation in the expected length of stay. The effect of ethnicity was perhaps most surprising given the existing literature [38, 40].

The association of sex on LOS is of some interest because it is known that males are more likely than females to suffer from asthma [15]; but all other things being equal, once admitted, females appear more likely to have a longer stay. Speculatively, this could be explained either by the fact that asthma events in females who are admitted are more severe than those events in males, or males recover more quickly once admitted, or there are unobserved social or system artefacts interacting with patients' sex to vary length of stay.

The remaining factors had “effect sizes” that were sufficiently large to suggest clinical significance. Perhaps unsurprisingly, diagnosis was significantly associated with length of stay. As a primary diagnosis, predominantly allergic asthma was associated with a reduced LOS compared with “asthma, unspecified”. Among the secondary diagnoses, co-morbidities of the lower respiratory tract were associated with an LOS up to 1.82. It was also interesting to observe the variation in the length of stay associated with the mode of admission. Admission based on a Consultant in an out-patient clinic, all other things being equal, was associated with an LOS 1.20 times longer, while General (Family) Practice admissions were associated with shorter stays (0.90). Explaining the former seems straightforward: a clinical specialist sees a patient and recognises someone in need of acute care, and those patients are on average more clinically acute than those who attend a hospital Accident & Emergency department without having first seen a doctor. Why admissions by the generalist medical practitioner, however, should on average require shorter stays than those who attend a hospital Accident & Emergency department directly is less clear.

It is well known that the numbers attending hospital Accident & Emergency departments vary by the day of the week [37]. The relationship between the day of the week and the length of stay is less frequently recorded, but has been observed [38]; and may relate to unobserved social and health service factors including bed occupancy rates or staffing levels.

From a health services perspective, large effects that involve relatively few patients are less important than smaller effect involving larger numbers of patients. Bearing this in mind, the explanatory variables of greatest health services interest appear to be sex (females have increased LOS), age (monotonically increasing LOS with increasing age), secondary diagnosis involving a disease of the lower respiratory tract (increased LOS), General practitioner referral (decreased LOS), day of the week (decreased LOS on Sundays), and year (monotonically decreasing LOS).

There are a number of limitations associated with this study. Methodologically, all that can be observed are associations, and the attribution of a causal link (just from these data) between the factors and the length of stay is impossible; although one would anticipate a straightforward causal link between, say, clinical diagnosis and length of stay. Notwithstanding the lack of clear causal pathways within the design of the research, the results can still be useful from a health services perspective, particularly when the results are interpreted within the wider body of knowledge about asthma.

One limitation of this study relates to the definition of asthma. Only ICD-10, J45 (*asthma*) coded admissions were in the data set, excluding J46 (*status asthmaticus*) coded admissions. This will affect the gerneralisability of the results. However, from previous research we know that the majority (90%) of asthma admissions in the UK are J45 coded [24]. Although J46 coded admissions tend, on average, to be more serious, there is considerable diagnostic overlap between the two codes, and in absolute numbers many J45 cases are more serious than J46 coded cases [24]. On balance, these results are likely to point to the correct direction of the relationship between the explanatory variables and LOS, although the specific estimates may need to be adjusted.

The second limitation is with definition of “length of stay” itself. There were a substantial number of admissions of zero days (17%). Zero days of admission, however, do not imply zero time or zero cost, and the measure does not reflect admissions of nearly 24 hours (underestimating LOS), or the high costs of monitoring and testing that occur early in the admission cycle. The full burden on hospitals is, therefore, likely to be higher than might be reflected here [20]. The interpretation of the results, therefore need to separate issues of bed occupancy from the total cost (and time distribution) of management and care. Nonetheless, appropriately weighted LOS results can be factored into a total cost analysis.

Finally, there are likely also to be unobserved factors, or factors for which HES data are unavailable, contributing to length of stay. Changes in the healthcare system, clinical management, and hospital culture for instance may explain some of the variation in the length of stay accounting for the annual decline, variations by day of the week, or sex differences. Capturing these factors when using routinely collected hospital data can be challenging, but the lurking limitation needs to be recognised.

Notwithstanding these limitations, the data are near to a complete record of asthma (J45) admission from 2001 to 2006 and the reported relationships do reflect what are in the data in that time period. For health services, this is useful. The demographic factors can be used directly to project hospital length of stay, and therefore project bed occupancy and some level of health service utilisation. The disparity between the length of stay associated with Consultant and General Practitioner admissions raise interesting clinical questions worthy of further investigation, and they are also informative for health services in projecting utilisation. Future research will ideally capture J46 admissions as well.

### Conclusion

Asthma admissions continue to be an important source of hospital admissions and account for a substantial number of bed days. Although the number of admissions has steadily risen over time, the average length of stay has steadily declined. Demographic, temporal, and diagnostic factors independently explain the variation in the length of stay. The identification of these factors is of clinical and health services interest; pointing to potential areas of future research, but also providing a basis for projecting health service utilisation.

## Supporting Information

Table S1 Summary statistics of asthma and related indicators of hospital admission records in London, 2001-2006.
(DOC)

Table S2 Multivariable base and reduced models of length of stay in asthma related hospital admissions in London, 2001-2006.
(DOC)
