# Supplementary material for: Asthma Length of Stay in Hospitals in London 2001–2006: Demographic, Diagnostic and Temporal Factors
Source: PLoS One. 2011 Nov 2;6(11):e27184. doi: 10.1371/journal.pone.0027184 (PMC3206938; doi:10.1371/journal.pone.0027184)
Supplement: Table S1 — Summary statistics of asthma and related indicators of hospital admission records in London, 2001-2006. (DOC) [file pone.0027184.s001.doc]

**Tables:**

**Asthma length of stay in hospitals in London 2001-2006: demographic, diagnostic and temporal factors**

**Ireneous N. Soyiri^1,2*^, Daniel D. Reidpath^2^, Christophe Sarran^3^**

**^1^**School of Public Health, University of Ghana, Accra, Ghana; **^2^**Global Public Health, School of Medicine & Health Sciences, Monash University, Sunway Campus, Malaysia; **^3^**Met Office, Fitzroy Road, Exeter EX1 3PB, United Kingdom

*Correspondence: [soyiriin@yahoo.com](mailto:soyiriin@yahoo.com)

**Table S1. Summary statistics of asthma and related indicators of hospital admission records in London, 2001-2006**

| **Characteristics** | **Frequency (%)** |
| --- | --- |
|
| **Sex** |  |
| Male | 27,329 (48.1) |
| Female | 29,449 (51.9) |
| **Age (years)** |  |
| Under 5 | 12,420 (12.4) |
| 5-14 | 10,700 (10.7) |
| 15-44 | 16,612 (16.6) |
| 45-59 | 7,029 (7.0) |
| 60-74 | 5,698 (5.7) |
| Over 75 | 4,309 (4.3) |
| **Ethnic Group** |  |
| White | 26,230 (46.2) |
| Black | 6,604 (11.6) |
| Asian | 6,382 (11.2) |
| Mixed/Other | 5,780 (10.2) |
| Not stated | 11,782 (20.8) |
| **Primary Diagnosis** |  |
| Asthma, unspecified | 53,637 (94.5) |
| Non-allergic asthma | 182 (0.3) |
| Mixed asthma | 54 (0.1) |
| Predominantly allergic | 2,905 (5.1) |
| **Secondary Diagnosis** |  |
| Other diseases of upper respiratory tract | 25,053 (44.1) |
| Influenza and Pneumonia | 692 (1.2) |
| Other acute lower respiratory infections | 6,256 (11.0) |
| Acute upper respiratory infections | 70 (0.1) |
| Chronic lower respiratory infections | 1,207 (2.1) |
| Lung diseases due to external agents | 1,519 (2.7) |
| Other diseases of respiratory system | 378 (0.7) |
| Other non-respiratory system diseases | 15,227 (26.8) |
| Missing Values | 6,376 (11.2) |
| **Method of Admission** |  |
| Accident and emergency services | 52,074 (91.7) |
| General Practitioner (GP) | 2,602 (4.6) |
| Bed bureau | 41 (0.1) |
| Consultants out patient clinic | 577 (1.0) |
| Other means | 1,484 (2.6) |
| **Day of the week** |  |
| Sunday | 5,369 (9.5) |
| Monday | 8,708 (15.3) |
| Tuesday | 9,740 (17.2) |
| Wednesday | 9,060 (16.0) |
| Thursday | 8,705 (15.3) |
| Friday | 9,163 (16.1) |
| Saturday | 6,033 (10.6) |
| **Meteorological Season** |  |
| Summer | 12,340 (21.7) |
| Spring | 13,453 (23.7) |
| Autumn | 16,800 (29.6) |
| Winter | 14,185 (25.0) |
| **Year of admission** |  |
| 2001 | 8,308 (14.6) |
| 2002 | 8,196 (14.4) |
| 2003 | 9,141 (16.1) |
| 2004 | 10,340 (18.2) |
| 2005 | 10,239 (18.0) |
| 2006 | 10,554 (18.6) |
| **Birth month** |  |
| January | 5,350 (9.4) |
| February | 4,384 (7.7) |
| March | 5,045 (8.9) |
| April | 4,644 (8.2) |
| May | 4,472 (7.9) |
| June | 4,591 (8.1) |
| July | 4,700 (8.3) |
| August | 4,637 (8.2) |
| September | 4,748 (8.4) |
| October | 4,803 (8.5) |
| November | 4,629 (8.2) |
| December | 4,775 (8.4) |
