# Supplementary material for: Asthma Length of Stay in Hospitals in London 2001–2006: Demographic, Diagnostic and Temporal Factors
Source: PLoS One. 2011 Nov 2;6(11):e27184. doi: 10.1371/journal.pone.0027184 (PMC3206938; doi:10.1371/journal.pone.0027184)
Supplement: Table S2 — Multivariable base and reduced models of length of stay in asthma related hospital admissions in London, 2001-2006. (DOC) [file pone.0027184.s002.doc]

**Tables:**

**Asthma length of stay in hospitals in London 2001-2006: demographic, diagnostic and temporal factors**

**Ireneous N. Soyiri^1,2*^, Daniel D. Reidpath^2^, Christophe Sarran^3^**

**^1^**School of Public Health, University of Ghana, Accra, Ghana; **^2^**Global Public Health, School of Medicine & Health Sciences, Monash University, Sunway Campus, Malaysia; **^3^**Met Office, Fitzroy Road, Exeter EX1 3PB, United Kingdom

*Correspondence: [soyiriin@yahoo.com](mailto:soyiriin@yahoo.com)

**Table S2. Multivariable base and reduced models of length of stay in asthma related hospital admissions in London, 2001-2006**

| **Characteristics** | **Base Model** | | | **Reduced Model** | | |
| --- | --- | --- | --- | --- | --- | --- |
|  | **^§^Ratio** | **[95% C.I.]** | | **^§^Ratio** | **[95% C.I.]** | |
| **Sex** |  |  |  |  |  |  |
| Male^#^ | 1.00 |  |  | 1.00 |  |  |
| Female | 1.11*** | 1.09 | 1.13 | 1.11*** | 1.09 | 1.13 |
| **Age (years)** |  |  |  |  |  |  |
| Under 5^#^ | 1.00 |  |  | 1.00 |  |  |
| 5-14 | 1.07*** | 1.04 | 1.11 | 1.07*** | 1.04 | 1.11 |
| 15-44 | 1.70*** | 1.66 | 1.75 | 1.70*** | 1.66 | 1.75 |
| 45-59 | 2.25*** | 2.18 | 2.32 | 2.25*** | 2.18 | 2.32 |
| 60-74 | 2.79*** | 2.70 | 2.88 | 2.79*** | 2.70 | 2.88 |
| Over 75 | 3.43*** | 3.31 | 3.55 | 3.43*** | 3.31 | 3.55 |
| **Ethnic Group** |  |  |  |  |  |  |
| White^#^ | 1.00 |  |  | 1.00 |  |  |
| Black | 1.05** | 1.02 | 1.08 | 1.05** | 1.02 | 1.08 |
| Asian | 1.01 | 0.99 | 1.04 | 1.02 | 0.99 | 1.04 |
| Mixed/Other | 1.00 | 0.97 | 1.03 | 1.00 | 0.97 | 1.03 |
| Not stated | 0.93*** | 0.91 | 0.95 | 0.93*** | 0.91 | 0.95 |
| **Primary Diagnosis** |  |  |  |  |  |  |
| Asthma, unspecified^#^ | 1.00 |  |  | 1.00 |  |  |
| Non-allergic asthma | 0.98 | 0.86 | 1.10 | 0.97 | 0.86 | 1.10 |
| Mixed asthma | 0.88 | 0.70 | 1.11 | 0.88 | 0.70 | 1.10 |
| Predominantly allergic | 0.83*** | 0.79 | 0.87 | 0.83*** | 0.79 | 0.87 |
| **Secondary Diagnosis** |  |  |  |  |  |  |
| Other diseases of upper respiratory tract^#^ | 1.00 |  |  | 1.00 |  |  |
| Influenza and Pneumonia | 1.82*** | 1.72 | 1.93 | 1.82*** | 1.72 | 1.93 |
| Other acute lower respiratory infections | 1.44*** | 1.41 | 1.48 | 1.44*** | 1.41 | 1.48 |
| Acute upper respiratory infections | 1.07 | 0.83 | 1.38 | 1.08 | 0.84 | 1.38 |
| Chronic lower respiratory infections | 1.33*** | 1.27 | 1.39 | 1.33*** | 1.27 | 1.39 |
| Lung diseases due to external agents | 1.02 | 0.97 | 1.08 | 1.02 | 0.97 | 1.08 |
| Other diseases of respiratory system | 1.69*** | 1.57 | 1.82 | 1.69*** | 1.57 | 1.82 |
| Other Non-respiratory system diseases | 1.24*** | 1.21 | 1.26 | 1.24*** | 1.21 | 1.26 |
| Missing Values | 1.02 | 0.98 | 1.05 | 1.02 | 0.98 | 1.05 |
| **Method of Admission** |  |  |  |  |  |  |
| Accident and emergency services^#^ | 1.00 |  |  | 1.00 |  |  |
| General Practitioner (GP) | 0.90*** | 0.86 | 0.93 | 0.90*** | 0.86 | 0.93 |
| Bed bureau | 1.15 | 0.89 | 1.48 | 1.14 | 0.89 | 1.48 |
| Consultants out patient clinic | 1.20*** | 1.12 | 1.28 | 1.20*** | 1.12 | 1.28 |
| Other means | 0.98 | 0.93 | 1.03 | 0.98 | 0.93 | 1.03 |
| **Day of the week** |  |  |  |  |  |  |
| Sunday^#^ | 1.00 |  |  | 1.00 |  |  |
| Monday | 1.32*** | 1.28 | 1.37 | 1.32*** | 1.28 | 1.37 |
| Tuesday | 1.37*** | 1.33 | 1.42 | 1.37*** | 1.33 | 1.42 |
| Wednesday | 1.30*** | 1.25 | 1.34 | 1.30*** | 1.25 | 1.34 |
| Thursday | 1.31*** | 1.26 | 1.35 | 1.31*** | 1.26 | 1.35 |
| Friday | 1.28*** | 1.24 | 1.33 | 1.28*** | 1.24 | 1.33 |
| Saturday | 1.09*** | 1.05 | 1.13 | 1.09*** | 1.05 | 1.13 |
| **Meteorological Season** |  |  |  |  |  |  |
| Summer^#^ | 1.00 |  |  | 1.00 |  |  |
| Spring | 1.05*** | 1.02 | 1.07 | 1.05*** | 1.02 | 1.07 |
| Autumn | 1.04*** | 1.02 | 1.06 | 1.04*** | 1.02 | 1.06 |
| Winter | 1.07*** | 1.05 | 1.09 | 1.07*** | 1.05 | 1.09 |
| **Year of admission** |  |  |  |  |  |  |
| 2001^#^ | 1.00 |  |  | 1.00 |  |  |
| 2002 | 0.98 | 0.95 | 1.01 | 0.98 | 0.95 | 1.01 |
| 2003 | 0.92*** | 0.89 | 0.95 | 0.92*** | 0.89 | 0.95 |
| 2004 | 0.83*** | 0.80 | 0.86 | 0.83*** | 0.80 | 0.86 |
| 2005 | 0.78*** | 0.75 | 0.80 | 0.78*** | 0.75 | 0.80 |
| 2006 | 0.71*** | 0.68 | 0.73 | 0.71*** | 0.68 | 0.73 |
| **Birth month** |  |  |  |  |  |  |
| January^#^ | 1.00 |  |  |  |  |  |
| February | 0.99 | 0.95 | 1.03 |  |  |  |
| March | 0.98 | 0.95 | 1.02 |  |  |  |
| April | 0.98 | 0.94 | 1.02 |  |  |  |
| May | 0.99 | 0.95 | 1.03 |  |  |  |
| June | 1.00 | 0.96 | 1.04 |  |  |  |
| July | 0.96* | 0.92 | 1.00 |  |  |  |
| August | 0.97 | 0.94 | 1.01 |  |  |  |
| September | 0.98 | 0.94 | 1.02 |  |  |  |
| October | 0.96 | 0.92 | 1.00 |  |  |  |
| November | 0.98 | 0.94 | 1.02 |  |  |  |
| December | 1.01 | 0.97 | 1.05 |  |  |  |
| *Akaike Information Criterion (AIC)* | *239405.5* | | | *239394.8* | | |

**^§^**Exponent of the coefficient, which is the expected change in log count for a one-unit increase in a “Characteristic”, interpreted as a ratio to the ^#^Reference category; C.I. Confidence Interval; * p<0.05; **p<0.01; *** p<0.001
